# Complete Genome Sequence of a Virulent Strain, *Streptococcus iniae* ISET0901, Isolated from Diseased Tilapia

**DOI:** 10.1128/genomeA.00553-14

**Published:** 2014-06-05

**Authors:** Julia W. Pridgeon, Dunhua Zhang, Lee Zhang

**Affiliations:** a Aquatic Animal Health Research Unit, USDA, ARS, Auburn, Alabama, USA; b Genomics and Sequencing Laboratory, Department of Entomology and Plant Pathology, Auburn University, Auburn, Alabama, USA

## Abstract

*Streptococcus iniae* ISET0901 is a virulent strain isolated in 2007 from diseased tilapia. Its full genome is 2,070,856 bp. The availability of this genome will allow comparative genomics to identify virulence genes important for the pathogenesis of streptococcosis caused by *S. iniae*, as well as possible immunogens for vaccine development.

## GENOME ANNOUNCEMENT

The Gram-positive bacterium *Streptococcus iniae* is a zoonotic pathogen that causes disease in both humans and fish (1–3). In aquaculture, *S. iniae* is a serious marine and freshwater fish pathogen causing significant economic losses (4, 5). Originally isolated from Amazon freshwater dolphin (*Inia geoffrensis*) in 1976 (6), *S. iniae* causes mortalities in >30 species of fish, including rainbow trout (*Oncorhynchus mykiss* [7]), barramundi (*Lates calcarifer* [8]), red drum (*Sciaenops ocellatus* [9]), flounder (*Paralichthys* spp. [10, 11]), and tilapia (*Oreochromis* spp. [12]). Strain *S. iniae* ISET0901 was cultured from diseased Nile tilapia (*Oreochromis niloticus*) during a disease outbreak in 2005 (13). Virulence studies (13) revealed that *S. iniae* ISET0901 is highly virulent to Nile tilapia. However, the virulence factors associated with the genome of *S. iniae* ISET0901 are unknown. Therefore, the complete genome sequence of *S. iniae* ISET0901 was determined in this study.

The genome of *S. iniae* ISET0901 was sequenced using the Illumina 1500 HiSeq platform. BioNumerics (Applied Maths) was used to assemble a total of 23,446,702 sequence reads, with an average length of 100.21 bp (estimated 1,134× coverage), using the complete genome of *S. iniae* SF1 (GenBank accession no. CP005941) as a reference. The assembled genome of *S. iniae* ISET0901 is 2,070,822 bp, with a G+C content of 36.8%. RNAmmer (14) predicted 12 copies of rRNA (4 copies of 5S RNA, 16S RNA, and 23S RNA), which is similar to that in the reference genome of *S. iniae* SF1 (15). The RAST server (16) predicted 1,982 coding sequences belonging to 303 subsystems, including 291 involved in carbohydrate catabolism, 149 in protein metabolism, 135 in the synthesis of amino acids and derivatives, 114 in cell wall and capsule synthesis, 95 in RNA metabolism, and 92 in DNA metabolism, including 77 in cofactors, vitamins, prosthetic groups, or pigments, 62 in nucleoside and nucleotide synthesis, 62 in fatty acid and lipid synthesis, 52 involved in virulence, disease, and defense, 47 in membrane transport, 37 in stress response, 31 in phosphorus metabolism, 26 in regulation and cell signaling, 7 in secondary metabolism, and 2 in motility and chemotaxis.

### Nucleotide sequence accession number.

The complete genome sequence of *S. iniae* ISET0901 was deposited at GenBank under the accession no. CP007586.
